# Y chromosome microdeletions in sperm DNA of infertile patients from Tamil Nadu, south India

**DOI:** 10.4103/0970-1591.44252

**Published:** 2008

**Authors:** Poongothai J. Sakthivel, Manonayaki Swaminathan

**Affiliations:** Centre for Computational Engineering and Networking, Amrita Vishwa Vidyapeetham, Kettukarar Thottam, Coimbatore-641105, Tamil Nadu, India; Emerald Heights College for Women, Ooty, Tamil Nadu, India

**Keywords:** AZoospermic factor, microdeletions, Y chromosome

## Abstract

**Context:**

Y chromosome microdeletions in infertile men of Tamil Nadu, South India.

**Aim:**

The paper assesses the association of Y chromosome microdeletions among infertile patients using several STS markers from each AZF (AZoospermic Factor) region and also aspires to determine whether the blood DNA microdeletion picture matches the semen DNA Yq microdeletion map.

**Materials and Methods:**

A total of 287 men, including 147 infertile men and 140 normozoospermic fertile controls were included for the study.

**Results:**

Screening 72 semen samples with the STS markers specific to AZF (a,b,c) regions showed Y chromosome microdeletions in 19 (12.9%) individuals. No deletion was observed in all the three AZF regions by screening 45 blood and 30 paired samples. None of the control men showed deletion for the 28 STS markers, which were used for the primary screening of the deletion of AZF a,b,c regions.

**Conclusion:**

Germ cell DNA can be analyzed for Yq microdeletions rather than blood DNA.

## INTRODUCTION

Infertility is a major health problem today affecting 10-15% of the couples[1] and in these couples, male factor infertility accounts for ∼50% of causes. The known causes of male infertility are quite numerous but can be grouped into a moderate number of major categories. It has been associated with several genetic and non-genetic conditions, such as hypogonadotrophic hypogonadism, testicular maldescence, structural abnormalities of the male genital tract, genital infections, impotency, previous scrotal or inguinal surgery, varicoceles, chronic illness, medication and exposure to chemicals.[2]

Genetic causes of infertility are an important etiological factor leading to irreversible partial or complete spermatogenetic arrest. Y chromosome microdeletions are a common molecular cause of spermatogenic failure. It has been identified in 9% of azoospermic, 7-10% of idiopathic severe oligozoospermic and 11.6% of severe oligoasthenozoospermic infertile patients.[3] The microdeletions that occur in the AZF [AZoospermic Factor] region of the long arm of Y chromosome affect genes that are involved in spermatogenesis. There are three recurrently deleted nonoverlapping subregions in the proximal, middle and distal Yq11 regions in the deleted interval designated as AZFa, AZFb and AZFc. Recently, a fourth region AZFd has been identified which lies between AZFb and AZFc.[4]

To date, most studies from India and the majority of studies worldwide have analyzed Yq microdeletions from DNA isolated from blood.[1,5–8] However, blood DNA might not be representative of sperm DNA, which is of different embryological origin. Sperm DNA might have a higher rate of deletions and DNA damage as a result of oxidative stress. Only few studies have analyzed sperm DNA for Y chromosome microdeletions.[9,10] Further, few markers confining to each AZF region were used in most of STS-based Y chromosome microdeletion studies. We, for the first time made an attempt to assess the association of Y chromosome microdeletions among Tamilian men of South India with azoospermia, oligozoospermia and oligoasthenozoospermia using several STS markers from each AZF region and also aimed to determine whether the blood DNA microdeletion picture matches the semen DNA Yq microdeletion map.

## MATERIALS AND METHODS

### Patients

Samples of blood were collected from 45 infertile men; semen samples from 72 infertile men; paired samples (blood and semen of same patient) from 30 infertile men at infertility clinics from Erode and Nilgiri districts of Tamil Nadu, South India. The patients were classified according to alterations detected in spermograms, based on the WHO technique,[11] into groups [Table 1] with oilgozoospermia (1-20 × 10^6^ spermatozoa /ml), asthenozoospermia (>60% of non-motile sperms), oligoasthenozoospermia, varicocele (normal count), azoospermia (no sperms in the ejaculate), teratozoospermia (>40% of abnormal sperms), and necrozoospermia (100% dead sperms). One hundred and forty normozoospermic (> 20 million sperms /ml of semen) males [90 blood, 30 semen, 10 paired samples] of proven fertility served as controls. Appropriate written informed consent according to protocols approved by the ethical committee of the center for Cellular and Molecular Biology, Hyderabad, India was obtained from all participants in this study. Only patients with an apparently normal 46,XY karyotype were included in this study. One patient′s scrotal Doppler study revealed bilateral significant varicocele with reflux on the left side.

**Table 1:** Geographical origin, ethnic/linguistic affiliation and spermogram of infertile and fertile men

Ethnic/linguistic affiliation	Geographic origin of samples Samples	Erode district				Nilgiri district				Total
				
		Blood	Semen	Blood and Semen (Paired)	Total	Blood	Semen	Blood and Semen (Paired)	Total	
Dravidian	Mild oligozoospermia	28	11	9	48	9	-	5	14	62
	Severe oligozoospermia	1	3	3	7	-	-	-	-	7
	Asthenozoospermia	3	38	8	49	-	-	1	1	50
	Oligoasthenozoospermia	1	20	2	23	-	-	1	1	24
	Varicocele	1	-	-	1	-	-	-	-	1
	Azoospermia	1	-	-	1	-	-	-	-	1
	Teratospermia	1	-	-	1	-	-	-	-	1
	Necrospermia	-	-	1	1	-	-	-	-	1
	Total	36	72	23	131	-	-	-	16	147
	Noormospermia	63	28	7	98	27	12	3	42	140

### DNA extraction and deletion mapping by polymerase chain reaction

DNA was extracted from 10 ml of peripheral blood[12] and from semen [13] using standard procedures. Polymerase chain reaction (PCR)-based studies for Y chromosome microdeletions on both infertile and control men were carried out using STS markers on the long arm of the Y chromosome. Initially the screening for AZF regions was done using 28 STS markers [Table 2]. To detect the heterochromatic region, sY160 was used in all cases. Individuals who had deletions in any of these STS markers were further analyzed by using the flanking STS markers[3,14] in order to map the deletion breakpoints.

**Table 2:** STS markers used for primary screening of AZF region

AZF region	STS markers
AZFa	sY746, sY740, sY86, sY741, sY84, sY745, DFFRY, DBY, sY615, sY743 sY709, sY744
AZFb	sY99, sY100, sY109, sY113, sY127, sY129, sY133, sY134, sY138, sY143
AZFc	sY152, sY146, sY156, sY255, sY254, sY158

Primers were synthesized using ABI392 Oligosynthesizer (Perkin Elmer, Foster City, Calif). Each marker was amplified separately in a 0.2 mL thin wall tube using an MJ Research Thermal Cycler (Waltham, MA, USA) with a female negative control sample. The PCR conditions used for STS markers were as follows: initial denaturation (94°C for 5 min) and subsequent denaturations (94°C for 45 sec) were the same for all the samples. Different annealing temperatures that were used for different STS markers were as follows: 53°C for 30 sec for sY84 and sY158; 55°C for 30 sec for sY86; 60°C for 1 min for sY746, sY740, sY741, sY745, DFFRY, DBY, sY615, sY743 sY709 and sY744; 57.4°C for 1 min for sY99, sY100, sY129 and sY133; 55°C for 1 min for sY109, sY138, sY143, sY146, sY152, and sY156; 50°C for 1 min for sY113; 60°C for 30 sec for sY254; 58°C for 30 sec for sY127, sY134 and sY255. Extension was 65°C for 1 min for sY127, sY134, and sY255, whereas for the other STS markers extension was 72°C for 2 min. Amplified PCR products were electrophoresed at 120V in 2% agarose gel impregnated with ethidium bromide at 5 µg/mL and visualized under UV light.

## RESULTS

### Primary screening

**Figure 1:**
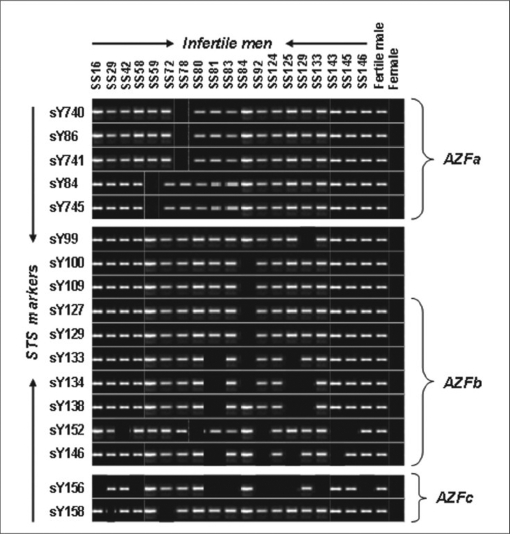
Gelimage showing PCR products of various sequence-tagged site (STS) markers representing the AZFa, AZFb, AZFc and DYZ regions

Screening 72 semen samples with the STS markers specific to AZF(a,b,c) regions showed Y chromosome microdeletions in 19 (five oligozoospermic, seven asthenozoospermic, seven oligoasthenozoospermic) individuals [Figure 1], which accounted for 12.9% of the total infertile men analyzed. The deletions that were detected in sperm DNA were confirmed thrice to rule out amplification failure. Surprisingly, no deletion was observed in all the three AZF regions by screening 45 blood and 30 paired (blood and semen) samples with STS markers. None of the control men showed deletion for the 28 STS markers, which were used for the primary screening of the deletion of AZFa, AZFb, and AZFc regions.

### Deletion mapping of Y chromosome

**Figure 2:**
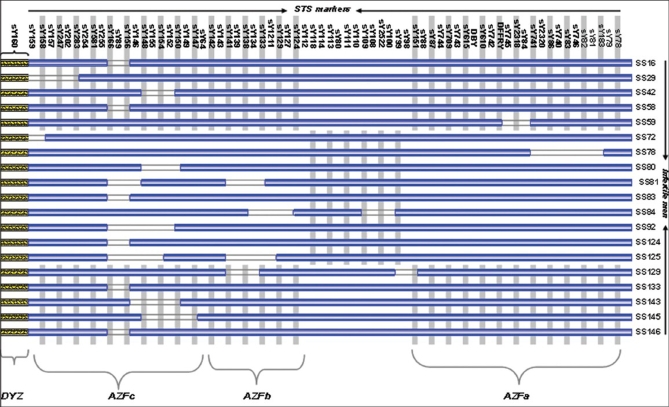
Y chromosome deletion map of infertile men generated by STS markers

**Figure 3:**
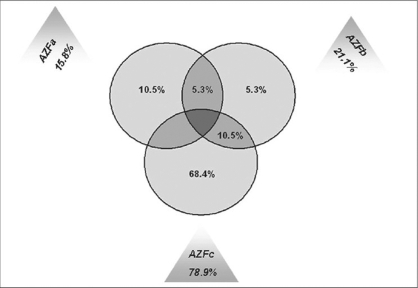
Venn diagram showing Y chromosome deletion in AZF regions

Micro and macrodeletions were noticed by further scrutinizing the deletions in these infertile men [Figure 2]. Deletion of the AZFa region alone was detected in two oligoasthenozoospermic men (SS59, SS78), which accounted for 10.5% of the total deletion [Figure 3]. One mild oligozoospermic man (SS84) showed deletion of the AZFb region whereas AZFab deletion was exhibited by SS129 severe oligospermic patient both of which accounted for 21.1% of the total deletions [Figure 3]. Deletion of AZFc alone was detected in 13 individuals (SS16, SS29, SS42, SS58, SS72, SS80, SS83, SS92, SS124, SS133, SS143, SS145, SS146), which accounted for 68.4% [Figure 3] of the total deletion. As a whole, deletion of AZFc region accounts for 78.9% [Figure 3] inclusive of AZFc region alone and AZFbc regions.

## DISCUSSION

For the first time, an attempt was made to evaluate the role of Y chromosome microdeletion in male infertility among Tamilians using copious STS markers from AZFa,b,c regions and also to determine whether the blood DNA microdeletion picture correlates with the semen DNA Yq microdeletion map. PCR-based STS analysis of 147 infertile men revealed micro- and macrodeletions on the Y chromosome in 19 individuals [Figure 1 and 2], accounting for 12.9% of the total infertile men analyzed. To our astonishment deletions were noticed only in the semen samples of infertile patients whereas no deletion was observed in blood and paired samples which correlates with the results of Mantas et al., 2007.[9] It was interesting to note that none of the azoospermic, necrozoospermic, teratozoospermic and varicocele sample showed deletion in any of the AZF regions. Previous studies revealed that the Y chromosome microdeletions were responsible for 3-13% of the infertile men.[7,8,15,16]

In this study, approximately one-sixth (15.8%) of the total Y chromosome deletion was in the AZFa region [Figure 3]. Of these 19 patients, deletion of the AZFa region alone was detected in two infertile men who were oligoasthenozoospermic (SS59, SS78), which accounted for 10.5% of the total deletion [Figure 3]. The size of the deletion in the AZFa region was very narrow (microdeletion) compared with the AZFb and AZFc regions. Neither of the individuals showed deletion of same STS marker. It was interesting that SS59 patient showed sY84 deletion and SS78 patient showed sY86 deletion [Figure 1], as these two markers were recommended for the diagnosis of the AZFa region by Simoni et al., 1999.[17] The further characterization of the microdeletions by extension analysis revealed that mild oligoasthenozoospermic (SS59) men showed deletion of middle region of AZFa and that of severe oligoasthenozoospermic men (SS78) confirmed proximal AZFa deletion [Figure 2]. Larger deletion of AZFa region in SS78 patient compared to SS59 might correlate with his severe phenotype. In general, frequency of deletion in the AZFa region was less compared with the AZFb and AZFc regions.[18,19] In most of the studies, deletion of the complete AZFa was found to be associated with SCOS.[18,20–22] This study demonstrated that even deletion of a narrow region on the AZFa region can cause SCOS as indicated by Thangaraj et al., 2003.[7]

Altogether, deletion of the AZFb region was involved in 21.1% of the total deletions, of which 5.3% was due to AZFb alone; another 5.3% was in association with AZFa and remaining 10.5% with AZFc [Figure 3]. Two different patterns of partial deletions [Figure 2] within the AZFb region were observed in one mild oligozoospermic (SS84) man in the patient group, which accounted for 1.4% (1/69) of the total oligozoospermic men included in this study. Arruda et al., 2007[23] reported 5% of AZFb deletion in oligozoospermic patients of the Brazilian population. Complete screening of the above patient for AZFb region revealed interesting results. Mild oligozoospermic patient (SS84) showed deletion of complete stretch of sY100, sY2522, sY108, sY109 and sY127, sY129, sY1211, sY133, sy134 STS markers. AZFab deletion was observed in severe oligozoospermic patient (SS129), but this individual showed deletion of one STS marker (sY151) in the AZFa region, whereas microdeletions of four STS markers (sY134, sY138, sy139, sY141) were observed in the AZFb region [Figure 2].

As a whole, deletion of the AZFc region accounts for 78.9% inclusive of AZFc region alone and AZFbc regions. Deletion of AZFc alone was detected in 13 individuals (SS16, SS29, SS42, SS58, SS72, SS80, SS83, SS92, SS124, SS133, SS143, SS145, SS146), which accounted for 68.4% [Figure 3] of the total deletion. In this study, minimum of total Y chromosome deletion was observed in AZFa and AZFb region whereas maximum deletion was seen in AZFc region. This is in agreement with the earlier studies which showed that the incidence of deletion in the AZFc region was high compared with the AZFa and AZFb regions.[24–27] Deletion of almost the same STS markers were found in SS143 and SS145 oligoasthenozoospermic patients; microdeletion of the same AZFc region in five asthenozoospermic men correlated with their phenotype [Figure 2]. Interestingly, two infertile men of different sperm anomalies showed deletion in both AZFb and AZFc regions [Figure 2], accounting for 10.5% of total deletion [Figure 3]. AZFbc deletion was observed by Fernando et al., 2006[28] with a frequency of 28.6% in the Sri Lankan population. Spermiograms of these infertile men showed that deletion of the AZFb and AZFc interval was associated with either asthenozoospermia, or severe oligozoospermia and insufficient production of mature sperm to enable reproduction.

Surprisingly, none of the infertile men from Nilgiri district showed deletion of AZF region which was evident from the results. This may be due to small sample size. None of the infertile and normal controls showed the deletion of the heterochromatic [Figure 2] region (DYZ). Although the Yq heterochromatic region predominantly consists of DYZ1 and DYZ2 classes of repeat sequences,[29] a few studies have shown its association with infertility and reproductive failure.

## CONCLUSION

The results of this work on the Tamilian population of South India highlights the need for larger more extensive studies to determine the frequency of deletions in blood and sperm DNA. Even though the percentage of Y chromosome microdeletions is lower in the selected population and it is noticed only in sperm DNA, in all cases opting for ART it is more applicable and necessary to analyze germ cell DNA than DNA isolated from blood for Yq microdeletions, since detection of these deletions has a prognostic value in predicting potential success of testicular sperm retrieval for assisted reproduction.
